# 

**Published:** 1874-03

**Authors:** 


☞Don't forget the one thing needful.☜

☞TO ADVERTISERS.—We send this number to several business houses whose advertisements we would be pleased to insert. Our circulation is among the very class of men who control the trade you desire, and we hesitate not to say that the RECORD is the best advertising medium ever published in the South for those who desire the trade of the Middle and Southern States, and who propose to conduct it in the regular way, viz: by keeping the active, every-day practitioner familliar with all the legitimate means and appliances used and approved in the cure of disease, that he may instruct his local druggist what and from whom to purchase. As journalists this is as far as we feel at liberty to go, as we do not propose the RECORD to be the medium of injury to either home or foreign druggists, but a medium for serving and doing good to all.

THE FIRST DAY OF APRIL is near at hand, and we desire all who have been contestants for the premiums offered, to send in their list, and the various premiums will be awarded *promptly* to those successfully claiming them. Send the name of any subscriber procured since the first of January, 1874.

Those who desire to compete for the premiums may secure subscribers by written appeals to their friends. They can say to them that, should they send in their names, to state to whom we are indebted for their subscriptions, and each name thus procured shall be counted in the contest for premiums. The money can be sent within sixty days after the reception of the first number. All subscriptions will be commenced with the January number, so long as they last. Send early, or they will be exhausted.

☞ Write your names, Post Office, County and State *plainly*. Be sure to say what Post Office you wish the RECORD sent.

☞ All communications, including remittances of money, etc., must be addressed to POWELL & GOLDSMITH.

Send money by check or Postal Order. Where these cannot be obtained, send at our risk, by registered letters.

As the editors work for "love and not money," spending every dollar made by the Journal on it—they hold no reserve fund with which to pay for articles. All friends of science and humanity are cordially invited to contribute articles for publication. The best we can do for our friends, is as follows:

All subscribers by *remitting* $3.30, will be entitled to Wood's Household Magazine, with a magnificent Chromo of the "Yosemite Valley"—worth, alone, several dollars.

All subscribers who will send two new subscriber (*besides his own name*,) shall receive one copy of *Powell's Pocket Formulary* or one copy of Wood's Household Magazine, with the superb Chromo of the "Yosemite Valley." Say plainly when remitting the money, which premium you desire.

All subscribers who will forward FIVE CASH SUBSCRIBERS (*besides his own name*,) will be entitled to one copy of the RECORD gratis: or, in lieu thereof, the Formulary and Wood's Household Magazine with Cromo. State clearly what you desire when the five subscribers are procured.

AS PRMIUMS, we will furnish *on the first of April next*, a splendid PORTABLE ELECTRO MNGNETIC MACHINE, Manufactured by the "Galvano Faradic Manufacturing Company," of New York, and worth $25.00, to any one sending us *the targest number of cash subscribers* over ten.

The one sending the next largest number over ten, will be entitled to the works of Sir. J. Y. Simpson, consisting of three volumes: Obstetrics and Gynœcology, Diseases of Women, Anæsthesia and Hospitalism.

The one sending the next largest number over ten, will receive "CODMAN & SHURTLIFF'S COMPLETE STEAM ATOMIZER FOR INHALATION.

The one sending the next largest number over *ten*, will be entitled to one copy of the RECORD, POWEL'S FORMULARY AND WOOD'S HOUSEHOLD MAGAZINE, with the large and magnificent CHROMO.

These instruments, books, etc., have been bought and await shipment to the successful persons, on the *first day of April next.* Go to work, friends, and make an effort to secure one of these splendid and useful premiums. This is the best we can do, and we pledge ourselves to comply, in every respect, to the terms above. ALL who do not procure the RECORD upon the several terms above given, are expected to PAY THE FULL SUBSCRIPTION PRICE.

☞ We can furnish a few more bound copies of the RECORD for 1873, at cost, $4.00 a volume.

LIST OF CASH PAYMENTS TO THE RECORD:

FOR 1873.

Dr H McGuffey, Louisiana.............. 2 50
Dr A B Wallace, Geo gia............ ... 2 50
Dr J C Howard, Florida.... .... .... 2 50
Dr C A Walker, Massachusetts.......... 2 50
Dr E M Bacon, Georgia - .............. 2 50
Dr Samuel T Looper, Georgia.......... 2 50
Dr W C Moore, Georgia .. ........... 2 50
Dr W L Lipscombe, Mississippi........ 2 50
Dr E Norton, South Carolina.......... 2 50
Dr O C Heery, Georgia.................. 2 50

FOR 1874.

Dr H M Talley, Georgia............. $2 50
Dr S H Hill, Alabama.... ........... 2 50
Dr J A Alexander Virginia.......... 2 50
Dr W H Rader, Virginia .. .... 2 50
Dr J M H Stover, Virginia.... ....... 2 50
Dr Pilson, Virginia.... ............. 2 50
Dr C P Sanders, Alabama............. 2 50
Dr T J Heard, Texas.... .......... 2 50
Dr R Fowler, Mississippi ............. 2 50
Dr H McGuffey, Louisiana............. 2 50
Dr A B Wallace, Georgia.............. 2 50
Dr T W Shockley, Mississippi.. .... 2 50
Dr Z T Young, Louisiana. .......... 2 50
Dr E W Parker, Ohio. ............... 2 50
Dr A W Schoht, North Carolina ..... 2 50
Drs Cox & Ellis, Mississippi.......... 2 50
Dr A P Harris, Mississippi........ .. 2 50
Dr E N Potts, Louisiana............. 2 50
Dr N G Tullis, Georgia........ .... . 2 50
Dr T S Roach, Alabama................ 2 50
Dr E A Anderson, North Carolina . .. 2 50
Dr George R Redwood, Mississippi.. . 2 50
Dr G D Hodge, Arkansas, .. . ... 2 50
Dr William R McCreight, Louisiana... 2 50
Dr John D Kline, Mississippi......... 2 50
Dr A H Smith, Mississippi,............. 2 50
Dr W R Perkins, Mississippi .. ....... 2 50
Dr B J Walker, Virginia . .......... 2 50
Dr D W Hammond, Georgia........... 2 50
Dr John Hardeman, Georgia........ .. 2 50
Dr J M Tullis, Georgia,... ..............2 50

FOR 1874.

Dr C A Walker, Massachusetts,. .. .... 2 50
Dr K H Davis, Georgia,... ............. 2 50
Dr B F Chapman, Georgia ............. 2 50
Dr S L Singletary, Louisiana... ....... 2 50
Dr L D Johnson, Georgia .......... 2 50
Dr H E Hurst, Alabama.................. 2 50
Dr J W Andrews, Alabama.. ........... 2 50
Dr W F Holt, Georgia. .................. 2 50
Dr W R Burgess, Georgia. .. .... 2 50
Dr William J Offutt, Louisiana ....... . 2 50
Dr Thomas A Cooke, Louisiana......... 2 50
Dr M E Demerest, Louisiana............ 2 50
Dr C Deaderick, Tennessee............. 2 50
Dr J M Kennedy, Tennessee ...... ... 2 50
Dr T M Shaw, Georgia........ ........ 2 50
Dr H B Lee, Georgia ............... .. 2 50
Dr J C Pastay, Georgia................ ... 2 50
Dr O C Heery, Georgia ................ 2 50
Dr J Starr, Georgia..... . ...... ... 2 50
Dr J L Cunningham, Texas.............. 2 50
Dr A Bulla, North Carolina.... ..... 2 50
Dr A L McCanless, North Carolina . .. 2 50
Dr C H Smith, Georgia............ ..... 2 50
Dr J M Taylor, Mississippi.. .... ..... 2 50
Dr Zeb T Murphy, Alabama............. 2 50
Dr W L Lipscombe, Mississippi........ 2 50
Dr B A Vaughan, Mississippi.......... .. 2 50
Dr J H Weir, Illinois... .. ........... 2 50
Drs Taylor & Price, Mississippi......... 2 50
Dr C C Heart, Georgia.... .......... 2 50
Dr M W Morton, Alabama............... 2 50
Dr J J Smith, North Carolina........... 2 50
Dr E Norton, South Carolina . .... ... 2 50
Dr J D Rush, Alabama............. ... 2 50
Dr Joseph Pogue, Illinois................ 2 50
Dr H T Wharff, Illinois........... .... 2 50
Dr B W Taylor, Illinois................... 2 50
Dr J L Martin, Oregon................... 2 50
Dr John Elsner, Colorado Territory..... 2 50
Drs Huggins & Prather, Mississippi. ... 2 50
Dr J B Dillard, Georgia.. .............. 2 50
Dr R Collings, Mississippi............... 2 50
Dr W W Wilkerson, Alabama........... 2 50

The above list of names are those who have paid for volume 73 and 74. If we have failed to credit any, they will do us the favor to inform us of the fact. March 23, 1874,

### ASSOCIATE EDITORS:

**GEORGIA.**

James A. Long, M.D.
W. L. M. Harris, M.D.
H. L Battle, M.D.
W. C. Musgrove, M D
L B. Bouchelle, M.D.
Thomas Burdell, M.D.
E. H. W. Hunter, M.D.
V. S Hitt, M.D.
J. E. Pope, M.D.
A. H. Brantley, M.D.
J. J. Knott, M.D.
J. C. Wisdom, M.D.
W. W. Leake, M.D.

VIRGINIA.

John H. Claiborne, M.D.
F. Horner, Jr., M.D.
R. J. Preston, M.D.
J. E. Lockridge, M.D.
J. S. Wharton, M.D.
Wm. O. Owen, M.D.
Sam'l P. Ripley, M.D.
Benj. Blackford, M.D.
E. A. Drewry, M.D.
Rob B. Tunstall, M.D.
W. F. Barr, M.D.
L. B. Anderson, M.D.
J. M. Lazzell, M.D.

ALABAMA.

J. C. Knox, M.D.
Geo. F. Taylor, M.D.
J. B. Barnett, M.D.
S. M. Hogan, M.D.
D. S. Hopping, M.D.
J. T. Searcy, M.D.
S. F. Hill, M.D.

NORTH CAROLINA.

J. R. Hughes, M.D.
S. S. Satchwell, M.D.
A. B. Pierce, M.D.
H. Kelly, M.D.
Geo. A. Foote, M.D.
E. A. Anderson, M.D
H. T. Bahnson, M.D.
Willis Alston, M.D.
Thos. F. Wood, M.D.
N. J. Pittman, M.D.
John W. Booth, M.D.

SOUTH CAROLINA.

E. T. McSwain, M.D.
G. M. Rivers, M.D.

TENNESSEE.

J. D. Tucker, M.D.

MISSISSIPPI.

C. B. Gallaway, M.D,
Edward Lea, M.D.
S. V. D. Hill, M.D.
E. T. Henry, M.D.
T. P. Lockwood, M.D.
Robert Kells, M.D.

KENTUCKY.

W. S. Taylor, M.D.
B. W. Stone, M.D.
Charles Kearns, M.D.

TEXAS.

S. M. Welch, M.D.
T. J. Heard, M.D.
W. A. Heard, M.D.

ARKANSAS.

A. D. Hodge, M.D.
J. K. Hodge, M.D.
A. W. Hobson, M.D.

OHIO.

J. Q. A. Hudson, M.D.

C. H. Tidd, M.D.

### CORRESPONDING EDITORS:

Ely Van DeWarker, M. D., New York.
C. C. F. Gay, M.D., New York,
*Surgeon of Buffalo General Hospital.*
M. H. Henry, M.D., New York,
*Surgeon to N. Y. Dispensary, Dep't of Venerial and Skin Diseases.*
Benjamin Howard, M.D., New York.
James Tyson, M.D., Philadelphia, Pa.
W. W. Keen, M.D. Philadelphia.
Geo. Strawbridge, M.D., Philadelphia.
Robert Robson, M.D., Indiana.
A. Patton, M.D., Indiana.
John H. Weir, M.D., Illinois.
Thomas J. Gallaher, M. D, Pennsylvania.
S. C. Busey, M.D., Washington, D. C.
Wm. R. Whitehead, M.D., Colorado Territory.

☞ The RECORD will be mailed between the 20th and 25th of each month.

